# Predictors of delayed hyponatremia after endoscopic transsphenoidal surgery for non-functioning pituitary adenomas

**DOI:** 10.3389/fendo.2025.1660531

**Published:** 2025-12-02

**Authors:** Xiaocheng Lu, Jingshan Sun, Yifan Wang, Yunfan Huang, Zhong Wang, Gang Chen

**Affiliations:** Department of Neurosurgery & Brain and Nerve Research Laboratory, The First Affiliated Hospital of Soochow University, Suzhou, China

**Keywords:** delayed postoperative hyponatremia, endoscopic transsphenoidal surgery, nonfunctioning pituitary adenoma, predictive factors, cerebrospinal fluid leak

## Abstract

**Background:**

Delayed postoperative hyponatremia (DPH) is a unique complication of endoscopic transsphenoidal surgery (TSS). Predictive factors for DPH following TSS in patients with nonfunctioning pituitary adenoma (NFPA) has not been well established. The aim of this study was to evaluate possible predictive factors related to DPH in patients with NFPA after TSS.

**Methods:**

We retrospectively analyzed 377 consecutive patients who underwent TSS for a NFPA between 2020 and 2024. The patients’ demographics, clinical features, as well as preoperative, intraoperative and postoperative data were collected, with statistical analysis of each factor performed.

**Results:**

Among the 377 patients with NFPA, DPH occurred in 112 (29.7%), including 24 (21.4%) had moderate hyponatremia and 13 (11.6%) had severe hyponatremia. Patients in DPH group had higher preoperative TSH level (3.01 vs 2.37 mIU/L, p=0.038), higher rates of intraoperative CSF leak (28.6% vs 16.6%, p=0.009) and postoperative meningitis (17.9% vs 7.5%, P = 0.004). In addition, hypertension (OR = 0.55, 95%CI 0.35-0.89, p=0.014), higher serum sodium of postoperative day 1 (POD 1) (OR = 0.88, 95%CI 0.83-0.94, p<0.001), and arginine vasopressin deficiency (AVP-D) (OR = 0.40, 95%CI 0.17-0.92, p=0.031) were linked to lower rates of DPH. In the multivariate analysis, hypertension (OR = 0.51, 95%CI 0.31-0.86, p=0.011), intraoperative CSF leak(OR = 2.60, 95%CI 1.40-4.81, p=0.002), serum sodium of POD 1(OR = 0.90, 95%CI 0.84-0.97, p=0.003), preoperative TSH(OR = 1.13, 95%CI 1.01-1.26, p=0.031) and meningitis(OR = 3.75, 95%CI 1.38-10.18, p=0.010) were identified as independent risk factors for DPH after TSS.

**Conclusion:**

This study identified the independent risk factors for delayed hyponatremia in patients with NFPA after TSS, including higher preoperative TSH level, intraoperative CSF leak, lower serum sodium of POD 1 and meningitis. However, patients with hypertension had a higher likelihood of avoiding DPH. It is recommended that DPH should be closely monitored after endoscopic TSS, especially in patients without hypertension. Furthermore, a multicenter prospective trial with an expanded sample size is warranted to investigate the etiology of DPH after endoscopic TSS.

## Introduction

Endoscopic transsphenoidal surgery (TSS) has become the mainstream surgical approach for sellar and parasellar lesions, especially pituitary adenomas, due to its minimally invasive nature and favorable outcomes (1–4). However, postoperative complications are still inevitable, which affect the postoperative recovery of pituitary adenoma patients (5–9). Delayed postoperative hyponatremia (DPH) remains a well-known complication following TSS that must be noticed. The incidence of DPH has been reported to be 9%-35% (10–14). DPH is defined as serum sodium <135 mmol/L after the third postoperative day, which usually occurs between postoperative days 4 and 10 (15).

Previous studies have pointed that DPH is a common cause of extended hospitalization and unexpected readmission after TSS for pituitary tumor (16, 17). The complication may be asymptomatic or may manifest as nausea, vomiting, altered mental state or seizures, and delayed treatment may result in severe complications. Thus, identification of predictive factors for DPH may facilitate early detection and postoperative management (18, 19). To date, several investigations have identified factors which may be associated with the development of DPH, including old age, lower preoperative serum sodium, larger tumor size and serum copeptin level (20, 21). However, these results are still controversial (22, 23). Due to the limited studies focusing on the risk factors for DPH in nonfunctioning pituitary adenoma (NFPA) and no consensus has been reached (12, 14), the present study was conducted as a retrospective review of our experience with NFPA patients following TSS to identify the predictive factors of DPH.

## Materials and methods

### Patients

The protocol of the present study was approved by the Institutional Review Board and Ethics committee of The First Affiliated Hospital of Soochow University (Suzhou, China). This study retrospectively analyzed clinical data of patients with pituitary adenoma who underwent TSS in The First Affiliated Hospital of Soochow University between January 2020 and March 2024. The inclusion criteria were as follows: (1) patients age ≥ 18 years; (2) patients with NFPA confirmed by clinical and pathological examination; (3) patients who underwent endoscopic TSS for pituitary adenomas; (3) with complete clinical data available. We excluded patients who were (1) operated trans-cranially; (2) pituitary adenomas not confirmed by pathology; (3) with other types of pituitary adenoma and (4) with NFPA combined with other pituitary lesions. All patients gave consent to use their clinical data for research purposes, and all data were anonymized.

### Data collection and definition

Demographic data (age and sex), laboratory test results, MRI scans and pathological data were retrospectively collected. Laboratory tests included preoperative serum sodium and postoperative serum sodium concentrations (the day after surgery and every 2–3 days subsequently) and preoperative and postoperative hormone levels (serum cortisol (ug/dL), prolactin (ng/mL), thyrotroph stimulating hormone (TSH) (mIU/L)), growth hormone (GH) (ng/mL), adrenocorticotropic hormone (ACTH) (pg/mL)). Delayed hyponatremia was defined as a serum sodium<135 mmol/L occurring on or after the postoperative day 3. The degree of hyponatremia was defined as follows: mild (130–134 mmol/l), moderate (125–129 mmol/l) and severe (< 125 mmol/l) (24). Postoperative meningitis was diagnosed as follows: fever (>38 °C), headache, stiff neck, meningeal signs, cranial nerve signs, or irritability; increased white blood cell count, increased protein level; and/or decreased glucose level in the CSF (25). The diagnostic criteria for arginine vasopressin deficiency (AVP-D) (previously called diabetes insipidus) were as follows: Urine production >300 mL/h for 3 consecutive hours and a corresponding urine specific gravity ≤ 1.005, in addition to at least one of the following: excessive thirst, serum osmolality >300 mOsmol/kg, or serum sodium>145 nmol/L (26).

Magnetic resonance imaging (MRI) (3.0 Tesla, Philips or Siemens) and computed tomography (CT) were performed to evaluate the tumor size and extent of resection in patients. The collected radiological features included optic nerve compression, maximum dimension of the tumor, intratumoral hematoma or cysts and Knosp grade. Cavernous sinus invasion was classified according to the Knosp classification (27). The Knosp grades 0–2 were defined as non-invasive adenomas and grades 3–4 as invasive adenomas.

### Perioperative management

All patients in this study underwent endoscopic TSS with the aid of a 0°, 30° and 70° high-definition endoscope (Karl Storz GmbH & Co.KG, Tuttlingen, Germany). The aim of the surgery was to remove the tumor as much as possible with preservation important structures, including the pituitary gland and pituitary stalk. Following operation, all patients received sodium chloride solution (0.9%) as maintenance fluid.

Patients who developed with AVP-D were treated with desmopressin acetate hydrate if necessary, and the dose of which was adjusted according to the fluid balance and serum sodium levels. Patients received an intravenous infusion of 200 mg hydrocortisone on postoperative day (POD) 0–1 and 100 mg on POD 2. Then patients had 50 mg oral hydrocortisone on POD 3–4 and 25 mg/day on POD 5-6. In addition, an appropriate hydrocortisone was continued in patients with permanent hypoadrenalism.

### Statistical analysis

All statistical data analysis and data visualization were performed with SPSS (version 23; IBM Corp.) and Sangerbox 3.0 (28). Mean and standard deviation were used to describe continuous data. Categorical variables were described as frequencies and percentages. Student t test was used to compare continuous variables and Chi-square (χ^2^) test or Fisher exact test was applied for categorical variables. After a univariant logistic regression analysis, the variables with p < 0.05 or clinically important factors were selected for the multivariate logistic analysis. In all cases, statistical significance was set at a 2-tailed P value less than 0.05.

## Results

From January 2020 to March 2024, a total of 377 patients with NFPA were included in this study. There were 194 females (51.5%) and 183 males (48.5%) and their mean age was 55.15 ± 12.75 years and 55.01 ± 11.62 years, respectively. The maximum diameter of tumor was 26.26 ± 10.39 mm, and most cases were macroadenoma (363/377, 96.3%). Among the 377 patients, 112 patients (29.7%) have developed DPH. The clinical characteristics of the overall patients are summarized in **Table 1**.

**Table 1:** Characteristics of the 377 patients with NFPA undergoing TSS.

Variable	Value
No. of patients	377
Age, mean ± SD, years	55.07 ± 12.16
Sex	
Female	194 (51.5%)
Age, mean ± SD, years	55.15 ± 12.75
Male	183 (48.5%)
Age, mean ± SD, years	55.01 ± 11.62
Co-morbidity	
Hypertension	147 (38.9%)
Diabetes mellitus	42 (11.1%)
Tumor size	
Microadenoma	14 (3.7%)
Macroadenoma	363 (96.3%)
Maximum diameter of tumor (mm)	26.26 ± 10.39
Tumor grade	
Invasive	69 (18.3%)
Non-invasive	308 (81.7%)
Compression of the optic chiasm	163 (43.2%)
Intratumoral hematoma or cysts	126 (33.4%)
Preoperative serum sodium (mmol/L)	140.11 ± 2.98
TSH (mIU/L)	2.56 ± 2.48
PRL (ng/mL)	20.84 ± 19.65
GH (ng/mL)	0.29 ± 0.57
ACTH (pg/mL)	43.72 ± 26.78
Cortisol (ug/dL)	10.62 ± 5.73
Intraoperative CSF leak	76 (20.2%)
Serum sodium for the first postoperative day	140.59 ± 4.06
Postoperative CSF leak	60 (15.9%)
Lumbar subarachnoid drainage	39 (10.3%)
DI	45 (11.9%)
Ki-67 expression ≥ 3%	167 (44.3%)
Meningitis	40 (10.6%)

ACTH, adrenocorticotropic hormone; CSF, cerebrospinal fluid; DI, diabetes insipidus; GH, growth hormone; NFPA, nonfunctioning pituitary adenoma; PRL, prolactin; TSH, thyrotroph stimulating hormone; TSS, transsphenoidal surgery.

**Figure 1:**
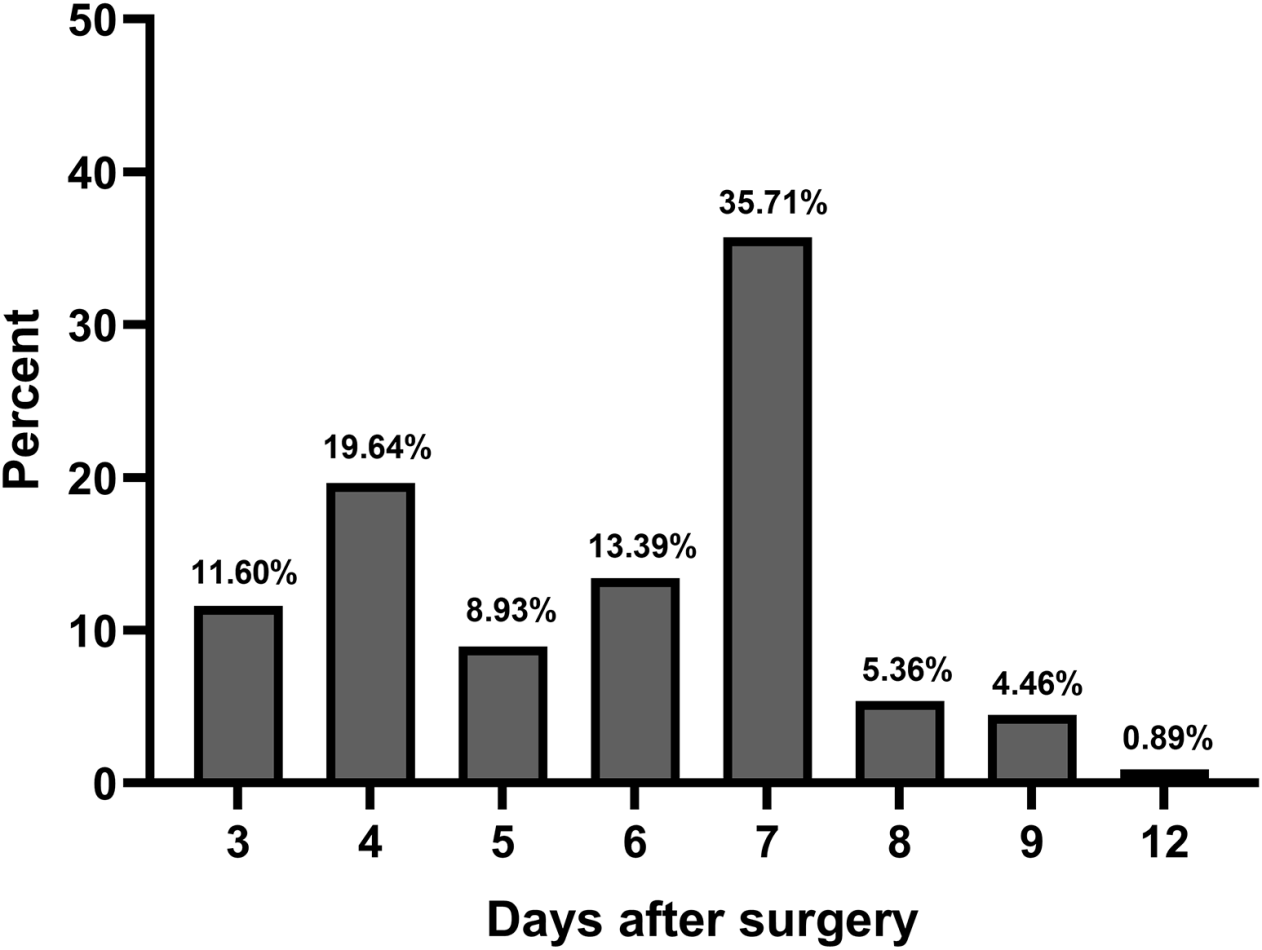
Time of occurrence of delayed hyponatremia.

Among 112 DPH patients, 75 patients (67.0%) had mild hyponatremia (132.30 ± 1.83 mmol/l), 24 (21.4%) had moderate hyponatremia (127.25 ± 1.31 mmol/l), and 13 (11.6%) had severe hyponatremia (121.52 ± 3.53 mmol/l). The timing of postoperative delayed hyponatremia occurrence was also analyzed, and the results indicated that delayed hyponatremia occurred mostly (40/112, 35.71%) on the seventh post−operative day (**Figure 1**). Patients with delayed hyponatremia (11.58 ± 5.05 days) had a significantly longer hospital stay than those with normonatremia (9.22 ± 4.96 days, p<0.001). We then compared the clinical, surgical characteristics and perioperative details between patients with DPH and those with normal serum sodium levels to identify potential predictors of DPH. The univariate analysis showed that the percentage of patients with hypertension was significantly lower in the DPH group (29.5%) than that in the normonatremia group (43.0%, p=0.014). In contrast, there were no remarkably differences between the two groups in age, BMI or diabetes mellitus. For factors related to MRI, there were no significant differences between two group in terms of maximum diameter of tumor, percentage of macroadenoma, invasiveness of tumor, compression of the optic chiasm and intratumoral hematoma or cysts.

We also compared the serum sodium levels before and after surgery between the two groups. The serum sodium levels on the first day after surgery was significantly lower in patients with DPH (139.37 ± 4.08 mmol/L) than in the patients with normonatremia (141.11 ± 3.89 mmol/L, p<0.001). However, there was no significant difference in term of the baseline serum sodium level between the patients with and without DPH. (DPH group vs normonatremia group, 140.05 ± 2.99 mmol/L vs 140.14 ± 2.99 mmol/L, p=0.775).

**Figure 2:**
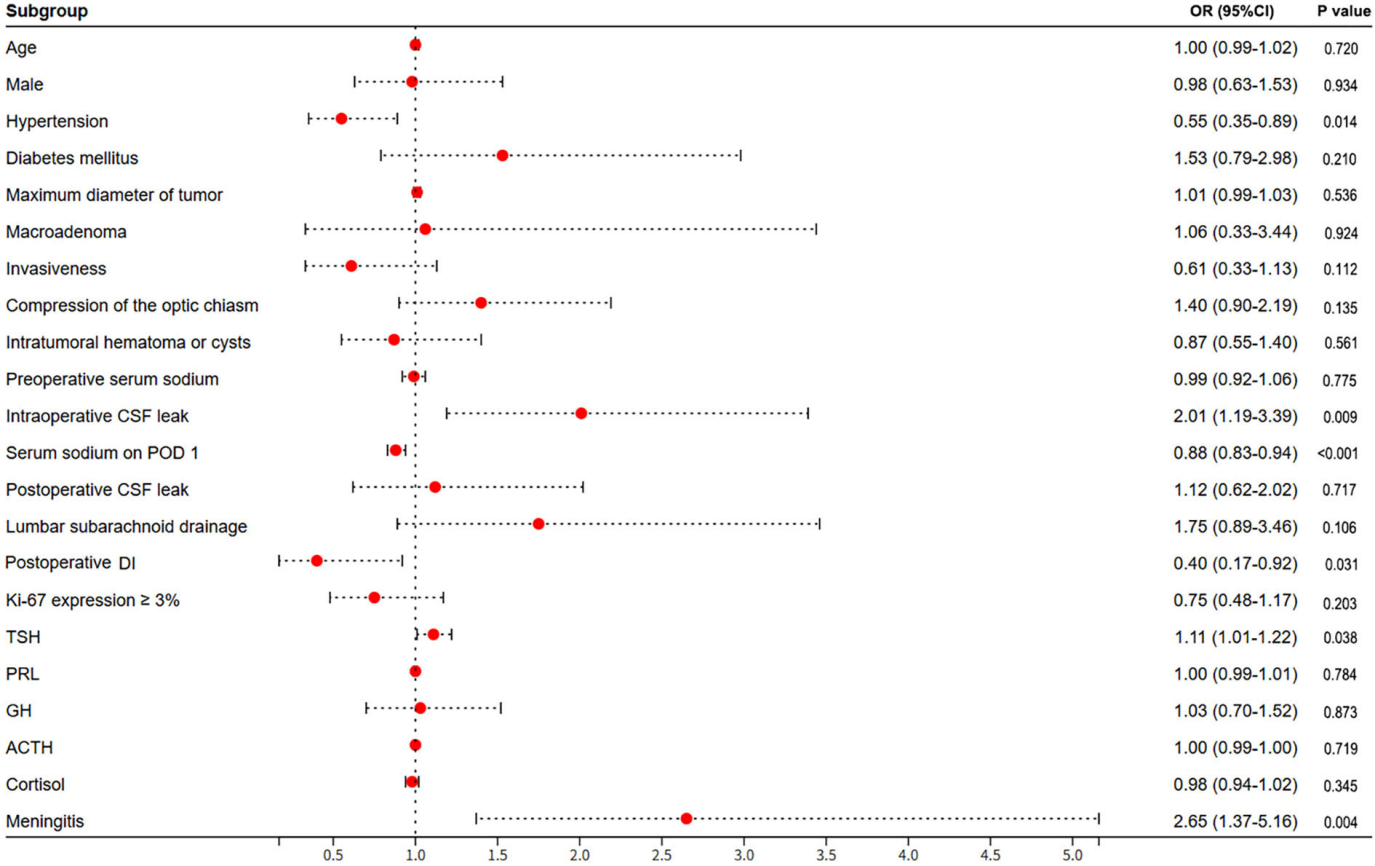
A univariate analysis was used to identify the risk factors for DPH after TSS in patients with NFPA. The red dots indicate OR values, and the error bars represent 95% CIs. DPH, delayed postoperative hyponatremia; TSS, transsphenoidal surgery; NFPA, nonfunctioning pituitary adenoma.

Regarding factors related to hormones, the result showed that the preoperative TSH level was significantly higher yet normal in the DPH group (3.01 ± 3.21 mIU/L), when compared with that in normonatremia group (2.37 ± 2.07 mIU/L, p=0.038). However, the preoperative levels of other hormones, such as PRL, GH, ACTH and cortisol, did not show a correlation with DPH. Next, factors related to surgery were also analyzed. We found that the percentage of intraoperative CSF leak was significant higher in patients with DPH (32/112, 28.6%) than that in patients with normonatremia (44/265, 16.6%, p=0.009). We also found that the occurrence of AVP-D was less common in patients with DPH (DPH group vs normonatremia group, 7/112, 6.3% vs 38/265, 14.3%, p=0.031). In addition, the presence of postoperative meningitis was also shown to be significantly correlated with DPH in the univariate analysis (DPH group vs normonatremia group, 20/112, 17.9% vs 20/265, 7.5%, p=0.004). However, other surgical related factors, such as postoperative CSF leak and lumbar subarachnoid drainage, did not show a correlation with DPH. The results of univariate analysis were shown in **Table 2** and **Figure 2**.

**Table 2:** Univariate analysis of factors related to postoperative-delayed hyponatremia.

Variable	Hyponatremia	Normonatremia	OR (95%CI)	P
No. of patients	112	265		
Age, mean ± SD, years	55.42 ± 12.31	54.93 ± 12.12	1.00 (0.99-1.02)	0.720
Sex				
Female	58 (51.8%)	136 (51.3%)	0.98 (0.63-1.53)	0.934
Male	54 (48.2%)	129 (48.7%)		
BMI	25.14 ± 3.29	24.53 ± 3.05	0.94 (0.88-1.01)	0.09
Co-morbidity				
Hypertension	33 (29.5%)	114 (43.0%)	0.55 (0.35-0.89)	0.014
Diabetes mellitus	16 (14.3%)	26 (9.8%)	1.53 (0.79-2.98)	0.210
Tumor size				
Macroadenoma	108 (96.4%)	255 (96.2%)	1.06 (0.33-3.44)	0.924
Microadenoma	4 (3.6%)	10 (3.8%)		
Maximum diameter of tumor (mm)	26.77 ± 10.84	26.05 ± 10.21	1.01 (0.99-1.03)	0.536
Tumor grade				
Invasive	15 (13.4%)	54 (20.4%)	0.60 (0.33-1.12)	0.112
Non-invasive	97 (86.6%)	211 (79.6%)		
Days of hospital stay	11.58 ± 5.05	9.22 ± 4.96		<0.001
Compression of the optic chiasm	55 (49.1%)	108 (40.8%)	1.40 (0.90-2.19)	0.135
Intratumoral hematoma or cysts	35 (31.3%)	91 (34.3%)	0.87 (0.55-1.40)	0.561
Intraoperative CSF leak	32 (28.6%)	44 (16.6%)	2.01 (1.19-3.39)	0.009
Serum sodium of POD 1	139.37 ± 4.08	141.11 ± 3.89	0.88 (0.83-0.94)	<0.001
Preoperative serum sodium (mmol/L)	140.05 ± 2.99	140.14 ± 2.99	0.99 (0.92-1.06)	0.775
Postoperative CSF leak	19 (17.0%)	41 (15.5%)	1.12 (0.62-2.02)	0.717
Lumbar subarachnoid drainage	16 (14.3%)	23 (8.7%)	1.75 (0.89-3.46)	0.106
DI	7 (6.3%)	38 (14.3%)	0.40 (0.17-0.92)	0.031
Ki-67 expression ≥ 3%	44 (39.3%)	123 (46.4%)	0.75 (0.48-1.17)	0.203
TSH (mIU/L)	3.01 ± 3.21	2.37 ± 2.07	1.11 (1.01-1.22)	0.038
PRL (ng/mL)	21.27 ± 19.40	20.66 ± 19.78	1.00 (0.99-1.01)	0.784
GH (ng/mL)	0.29 ± 0.64	0.28 ± 0.54	1.03 (0.70-1.52)	0.873
ACTH (pg/mL)	42.95 ± 27.24	44.04 ± 26.63	1.00 (0.99-1.00)	0.719
Cortisol (ug/dL)	10.20 ± 5.26	10.80 ± 5.91	0.98 (0.94-1.02)	0.345
Meningitis	20 (17.9%)	20 (7.5%)	2.65 (1.37-5.16)	0.004

ACTH, adrenocorticotropic hormone; CSF, cerebrospinal fluid; DI, diabetes insipidus; GH, growth hormone; POD, postoperative day; PRL, prolactin; TSH, thyrotroph stimulating hormone.

**Figure 3:**
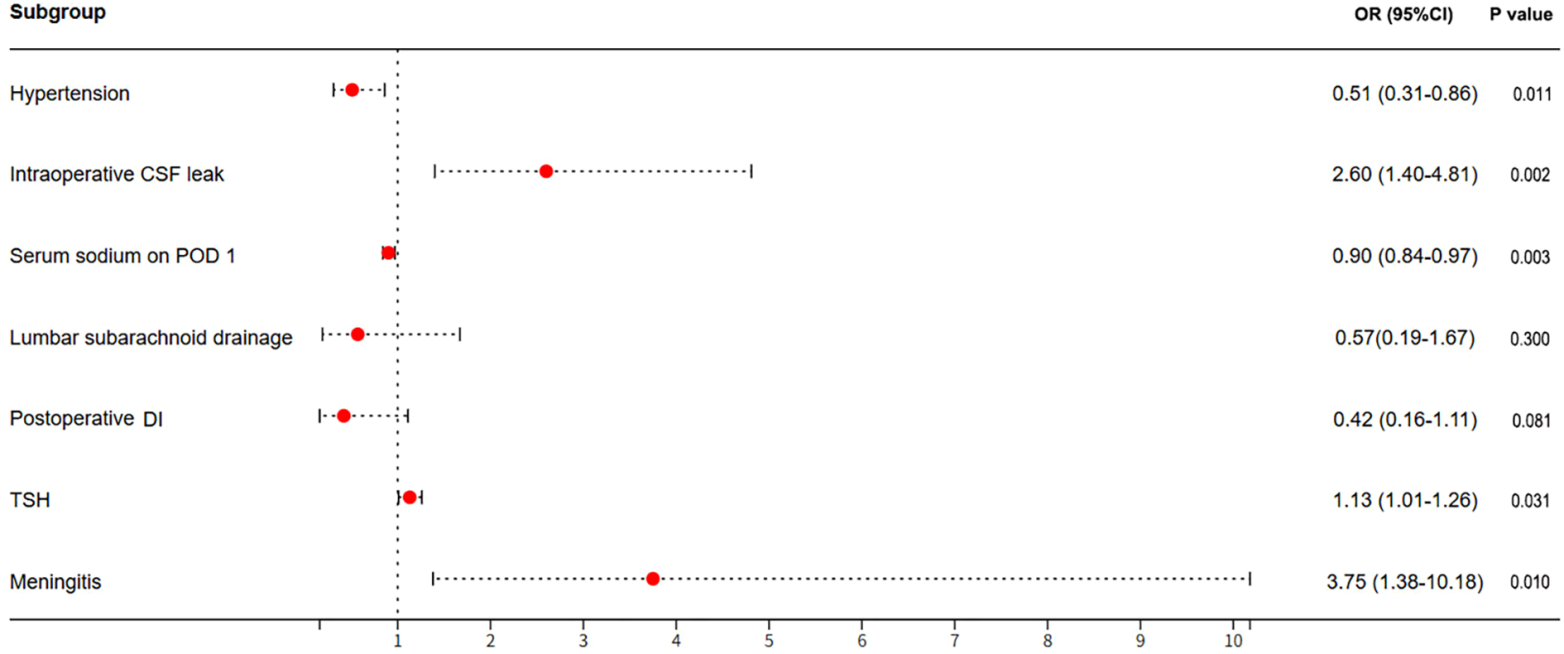
A multivariate logistic regression analysis was performed to identify the risk factors for DPH after TSS in patients with NFPA. The red dots indicate OR values, and the error bars represent 95% CIs. DPH, delayed postoperative hyponatremia; TSS, transsphenoidal surgery; NFPA, nonfunctioning pituitary adenoma.

Further, a multivariate logistic regression analysis to verify the predictors detected by the univariate analysis, such as hypertension intraoperative CSF leak, serum sodium of postoperative day 1, p AVP-D, as well as postoperative meningitis (**Table 3**; **Figure 3**). The results showed that the preoperative higher TSH level tended to be associated with DPH (odds ratio, 1.13; 95%CI, 1.01-1.26; p=0.031). In addition, intraoperative CSF leak (odds ratio, 2.60; 95%CI, 1.40-4.81; p=0.002) and postoperative meningitis (odds ratio, 3.75; 95%CI, 1.38-10.18; p=0.010) were also significantly associated with the development of DPH. In contrast, patients with hypertension (odds ratio, 0.51; 95%CI, 0.31-0.86; p=0.011) and high serum sodium levels POD 1 (odds ratio, 0.90; 95%CI, 0.84-0.97; p=0.003) were less likely to develop DPH after TSS. Moreover, patients with DI were likely to have a lower occurrence of DPH, however it was not significant in the multivariate analysis. (odds ratio, 0.42; 95%CI, 0.16-1.11; p=0.081).

**Table 3:** Multivariate analysis of factors related to postoperative-delayed hyponatremia.

Variable	Hyponatremia	Normonatremia	OR (95%CI)	P
Hypertension	33 (29.5%)	114 (43.0%)	0.51 (0.31-0.86)	0.011
Intraoperative CSF leak	32 (28.6%)	44 (16.6%)	2.60 (1.40-4.81)	0.002
Serum sodium of POD 1	139.37 ± 4.08	141.11 ± 3.89	0.90 (0.84-0.97)	0.003
Lumbar subarachnoid drainage	16 (14.3%)	23 (8.7%)	0.57 (0.19-1.67)	0.300
DI	7 (6.3%)	38 (14.3%)	0.42 (0.16-1.11)	0.081
TSH	3.01 ± 3.21	2.37 ± 2.07	1.13 (1.01-1.26)	0.031
meningitis	20 (17.9%)	20 (7.5%)	3.75 (1.38-10.18)	0.010

CSF, cerebrospinal fluid; DI, diabetes insipidus; POD, postoperative day; TSH, thyrotroph stimulating hormone.

## Discussion

DPH is one of the common complications in patients with pituitary adenomas following TSS. It is difficult to recognize, and its clinical course is difficult to predict. Although most patients with mild DPH are asymptomatic or non-specific symptoms, such as headache, nausea and vomiting. Severe hyponatremia can cause altered mental state, seizures, prolonged hospitalization, and delayed treatment may even lead to death. Therefore, predicting the incidence of DPH after TSS and early treatment are crucial. The treatment for delayed hyponatremia mainly depends on the severity and potential causes of hyponatremia. Fluid intake should be restricted in patients with DPH caused by SIADH (29). If cerebral salt wasting syndrome is the cause, then sodium salt and blood volume should be supplemented (30). In addition, exogenous mineralocorticoids have also been used to treat hyponatremia in patients with CSW (31) and of hyponatremia related to secondary adrenal insufficiency (32). Continual monitoring of electrolytes should be conducted until the serum sodium concentration has stabilized under stable treatment (33). Additionally, the risk of central pontine myelinolysis caused by rapid correction of hyponatremia should also be given priority attention. It is recommended to limit the increase in serum sodium concentration to a total of 10 mmol/L during the first 24 hours and 18 mmol/L during the first 48 hours until the serum sodium concentration reaches 130 mmol/L (24, 34).

In this retrospective study, we examined the incidence of DPH after endoscopic TSS for NFPA and aimed to identify the risk factors for DPH. Of 377 patients who underwent surgical procedure, DPH occurred in 29.7% of patients, which was comparable to the incidences reported in previous studies (9%-35%) (20, 21, 23, 35, 36). The results showed that hypertension was negatively correlated with the incidence of DPH. In contrast, intraoperative CSF leak, higher level of preoperative TSH, postoperative meningitis and lower serum sodium level of POD 1 were significantly associated with an increased occurrence of DPH.

The pathophysiology of delayed hyponatremia after TSS could be the result of either syndrome of inappropriate antidiuretic hormone secretion (SIADH), cerebral salt wasting syndrome, adrenocortical insufficiency, exogenous desmopressin administration or volume overload (33, 37, 38). In general, the SIADH has been reported as the most common cause of DPH, the mechanism of which is intraoperative trauma to the neuro-hypophysis that contributes to uncontrolled arginine vasopressin (ADH) release, leading to water retention (38). The clinical significance of DPH has gradually gained the attention among clinicians. Michael et al. reported that DPH was the most common cause of unplanned 30-day readmission in patients with pituitary adenoma after TSS (35, 39). Thus, identification of reliable predictive factors for DPH is important for guiding postoperative management and avoiding serious complications.

Several studies have indicated a correlation between DPH and some clinical characteristics, such as age, sex, pre-operative hypopituitarism and the size of adenoma, however, the results were controversial. Age (old and young) and gender have been identified as risk factors of DPH in several studies. Mo et al. reported that 48 years old was the cut-off age for predicting DPH (40). Tomita et al. reported 55 years as the cut-off age, and Kinoshita et al. reported a cut-off age of 60 years (41, 42). However, the results were controversial, Sorba et al. reported that younger age was an independent risk factor for SIADH (43). Females were reported to be more susceptible to DPH after surgery than males (33), whereas, Rajaratnam et al. showed that men were at higher risk of delayed postoperative hyponatremia (44). In the current study, we found that age and sex did not predict the occurrence of DPH in patients with NFPA after TSS.

Preoperative hormone levels were also investigated in the previous studies. In this study, we found that higher yet normal TSH level was statistically associated with DPH, which was similar to the results of a recent study (41). The TSH level was also shown to be significantly higher in patients with hyponatremia, whereas the mechanism remained unclear (45, 46). A recent study showed that preoperative serum hyperprolactinemia was also an independent risk factor for DPH patients with NFPA (14). However, in this study, we did not found other preoperative serum hormone levels to be reliable predictors of DPH, including the levels of PRL, GH, ACTH and cortisol. To date, a few studies have focused on the relationship between hypertension and delayed hyponatremia after pituitary surgery. One study involving 137 patients found that patients with hypertension had a tendency to avoid of delayed hyponatremia after pituitary surgery (12), while another study showed opposite results (41). In the present study, the result indicated that hypertension might be a protect factor of delayed hyponatremia, which might be due to the use of diuretics (47). However, the exact mechanism of this association with hypertension and DPH remains unclear, and further investigations are needed to validate these findings and explore the underlying mechanisms.

DPH usually occurs approximately 4–10 days after surgery, low sodium concentration on POD 1–2 has been reported as the predictive factor of DPH (13). In the present study, the serum sodium concentration on POD 1 was also identified as a significant risk factor of DPH after endoscopic TSS, which was comparable to the results reported by Krogh et al. (48). This lower serum sodium level which occurred on POD 1–2 might be caused by the abnormal secretion of ADH postoperatively, although the serum sodium concentration remains within the normal range (36). However, preoperative serum sodium level had no association with the occurrence of DPH.

Postoperative AVP-D has been identified as a predictive factor of DPH after TSS surgery. Zada et al. reported an increased incidence of DH in patients with AVP-D (15). In this study, univariate analysis results showed that patients with AVP-D were likely to have a lower risk of DPH, although it was not statistically significant in the multivariate analysis. Additionally, whether the size and invasiveness of tumors were associated with delayed hyponatremia remains controversial in previous studies (40). The results of this study did not support relationships between tumor size or Knosp grade and delayed hyponatremia.

We also found that intraoperative CSF leak and meningitis were independent risk factors for DPH after surgery, which were similar to a previous study (12). Fever caused by meningitis might lead to dehydration, triggering ADH secretion and necessitating hydration therapy. A rebound effect caused by such hydration treatment might be a potential mechanism for meningitis associated with DPH. A recent study indicated that hyponatremia was significantly more common in those patients with a lumbar subarachnoid drainage (49). Sodium depletion might occur following a few days of drainage as the sodium concentration in the CSF was almost 147.1 mmol/L (50). Thus, lumbar subarachnoid drainage was also included in the multivariate analysis in this study. However, in this study, we did not find the correlation between lumbar subarachnoid drainage and DPH.

Several limitations to this study should be considered. First, it was a retrospective study, which might introduce inherent bias. Second, the DPH was detected during hospitalization, thus we could not analysis the patients developing DPH after discharge. Third, although we investigated the associations of tumor size and Knosp grade with the risk of DPH, the tumor location may also influence the occurrence of DPH (51, 52). Finally, a single-center study might introduce potential bias to the results. Therefore, a multicenter prospective trial with larger sample size was warranted to investigate the DPH after TSS in the future.

## Conclusion

This study identified the independent risk factors for delayed hyponatremia in patients with NFPA after TSS, including intraoperative CSF leak, lower serum sodium of POD 1 and meningitis. However, patients with hypertension showed a tendency to avoid DPH. Therefore, attention should be given to non-hypertensive patients with intraoperative CSF leak, lower serum sodium of POD 1 or meningitis after TSS for possible occurrence of DPH.

## Data Availability

The data analyzed in this study is subject to the following licenses/restrictions: The raw data supporting the conclusions of this article will be made available by the authors, without undue reservation. Requests to access these datasets should be directed to XL, xiaochenglu@suda.edu.cn.
